# The rise of the Himalaya enforced the diversification of SE Asian ferns by altering the monsoon regimes

**DOI:** 10.1186/1471-2229-12-210

**Published:** 2012-11-09

**Authors:** Li Wang, Harald Schneider, Xian-Chun Zhang, Qiao-Ping Xiang

**Affiliations:** 1 State Key Laboratory of Systematic and Evolutionary Botany, Institute of Botany, the Chinese Academy of Sciences, Beijing, 100093, China; 2 Department of Botany, The Natural History Museum London, London, SW7 5BD, UK

**Keywords:** Diversification pattern, East Asian monsoon, Himalaya, LASER, *Lepisorus*

## Abstract

**Background:**

The rise of high mountain chains is widely seen as one of the factors driving rapid diversification of land plants and the formation of biodiversity hotspots. Supporting evidence was reported for the impact of the rapid rise of the Andean mountains but this hypothesis has so far been less explored for the impact of the “roof of the world”. The formation of the Himalaya, and especially the rise of the Qinghai–Tibetan Plateau in the recent 20 million years, altered the monsoon regimes that dominate the current climates of South East Asia. Here, we infer the hypothesis that the rise of Himalaya had a strong impact on the plant diversity in the biodiversity hotspot of the Southwest Chinese Mountains.

**Results:**

Our analyses of the diversification pattern of the derived fern genus *Lepisorus* recovered evidence for changes in plant diversity that correlated with the strengthening of South East Asian monsoon. Southwest China or Southwest China and Japan was recovered as the putative area of origin of *Lepisorus* and enhancing monsoon regime were found to shape the early diversification of the genus as well as subsequent radiations during the late Miocene and Pliocene.

**Conclusions:**

We report new evidence for a coincidence of plant diversification and changes of the climate caused by the uplift of the Himalaya. These results are discussed in the context of the impact of incomplete taxon sampling, uncertainty of divergence time estimates, and limitations of current methods used to assess diversification rates.

## Background

The origin of the uneven distribution of plant diversity, in particular with biodiversity hotspots [1], has been increasingly explored by employing phylogenetic methods to reconstruct the history of selected lineages. The majority of these studies addressed the origin of the Andean (e.g., [2-4]), Great Cape region (e.g., [5,6]) and Madagascan (e.g., [7,8]) biodiversity hotspots. These studies not only assembled evidence for the evolution of biodiversity of these particular hotspots but also laid out the foundation for a generalized theory on the history of biodiversity hotspots by classifying the attribution of diversification events into mature and rapid radiations [9]. Besides some shared features, the unique geographic and historical aspects, e.g. isolation by distance, relative constant climate, and major orogenic events, resulted in unique patterns of biodiversity assembly in these hotspots.

Current macro-evolutionary studies have given remarkably little attention to the origin of the biodiversity hotspots in Southeast (SE) Asia such as the southwest Chinese mountain region [1]. However, the origin of the later biodiversity hotspot is widely attributed to the rise of the Himalaya and the subsequent formation of the Qinghai-Tibetan Plateau (QTP), which is frequently referred as “the roof of the world” (e.g., [10-16]). In this respect, this hotspot is comparable to the Andean biodiversity hotspot attributed to the rise of the Andes (e.g., [2-4]). The rise of the Himalaya was onset by the collision of the Indian plate with the Asian plate around 50-55 million years ago (Ma) and is seen widely as one of the largest orogenic events in the history of Earth with dramatic implications on the climates of Central and East Asia. Despite some controversy about the exact timing of the uplift of the Himalaya [17-22], geological estimates date the start of this process unequivocally within the late Tertiary/mid-Miocene (ca. 15-10 Ma) [23] , and episodes of uplift probably continued throughout the late Pliocene (ca. 3 Ma) and well into the Quaternary [24-26]. Major impacts of the latest uplift of the Himalaya on the evolution of life in Southwest China has been suggested because the steep mountain ranges not only caused the formation of desert-like habitats in central Asia, but also changed drastically the East Asian monsoon [21,22,27-29]. The exact climate history of these events is still subject of controversy [18,21], but existing evidence suggests an initiation of the East Asian monsoon in the late Oligocene followed by several periods of strengthening in the Miocene (e.g., ~15 Ma & ~ 8 Ma) and a putative abrupt strengthening in the Pliocene / Pleistocene periods (~3 Ma) [30,31]. Various scenarios have been discussed [17,18,20-22,28] including the hypothesis of an abrupt weakening of the East Asian summer monsoon around 7.5 Ma [32]. Despite the ambiguity concerning the exact pattern, consensus exists concerning an onset of the current monsoon regimes in the Miocene and its fluctuation during the late Miocene, Pliocene and Pleistocene. The resulting changes in the amount and seasonal distribution of precipitation have likely supported a high rate of diversity turnovers including radiations of various plant lineages that compose the unique biodiversity of this region today.

Ferns are well suited to address the impact of climatic change modulating not only the maximum amount of precipitation but also its annual distribution. Ferns contribute a substantial component of the extant plant diversity in this region of the world. Recent studies rejected long-held assumptions of antiquity of fern diversity and established the post-Cretaceous origin of the majority of ferns [33-35]. Fern diversity is positively correlated with precipitation and most of the extant diversity is found in tropical mountain ranges [36]. In this context, the high fern species diversity in the SE Asia is highly remarkable and may reflect the combination of favorable climatic conditions, landscape structures and geographical history of this region [37]. A further advantage of ferns is their ability of dispersal and reproduction independent from biological interactions, which have a strong influence on the lineage history of most angiosperms [38]. Here, we focus on the derived fern genus *Lepisorus* that includes mainly epiphytic or saxicolous plants. The genus shows an accumulation of its species diversity in this region and some species display an adaptation to seasonality of the precipitation by having a leafless dormancy phase [39,40].

Hypotheses concerning the diversification rate changes are now regularly studied using DNA-based phylogenies despite limitations of the analytical approaches (e.g., [41-45]). Taxonomic sampling appears to be one of the major sources of errors, especially when the sampling covers <80% and/ or is non-random [42]. Thus, we sampled here more than 90% of the currently recognized species diversity of *Lepisorus* adopting the up-to-date classification [46-48], avoided a bias by sampling mainly deeper nodes and used simulations to assess the impact of incomplete taxon sampling. To our advantage, the taxonomy and phylogeny of the genus has been addressed in several recent studies and we consider here the most recent treatments [46-51]. These studies provided us with a rather comprehensive understanding of the species diversity and phylogenetic structure of this genus, although some uncertainty still exists concerning the number of species in this lineage that originated by primary speciation (speciation via cladogenese). Relatively little evidence for secondary speciation (speciation via polyploidy) was reported for this genus [52]. Rates of secondary speciation cannot be estimated using the established phylogenetic methods of diversification rate analyses because these analyses incorporate only the rate of primary speciation, meaning speciation by lineages splits via cladogenese events. These issues need to be considered in the interpretation of the results on the diversification of plants in which polyploidization is frequent [53]. We also consider the impact of phylogenetic and related uncertainties. Thus, we used several methods to study the rate of diversification [54-56] and employed Bayesian Dispersal-Vicariance analyses [57,58] to reconstruct ancestral ranges of lineages.

## Results

### Divergence time estimates

**Figure 1:**
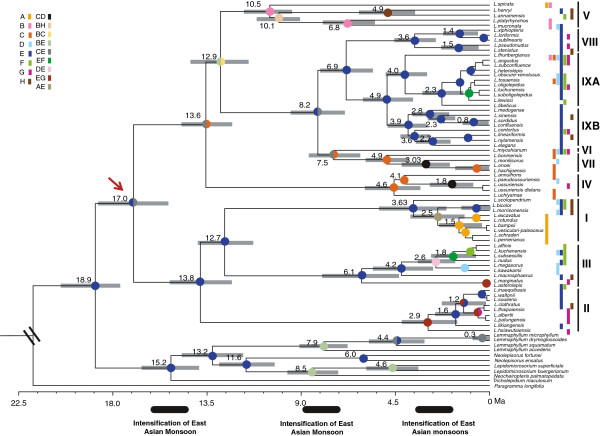
**Combined chronogram and biogeographic analysis of the paleotropical genus *Lepisorus*.** The tree is obtained from the BEAST analysis, with branches proportional to absolute age (in millions of years) calculated from median branch length of 10,000 Bayesian trees. The tree was calibrated at the most basal node (*Paragramma* sister to the remaining lepisoroid ferns) with 19.6 Ma (based on Schuettpelz and Pryer, 2009; Schneider et al., 2010) as a log-normal distributed calibration, which is indicated with the sign “\\”. The well-resolved nine clades are indicated as I – IX (Wang et al. 2010a). Node charts show the relative probabilities of alternative ancestral distribution obtained by statistical dispersal-vicariance analysis (S-DIVA) optimization over the 10,000 Bayesian trees. Present ranges for each species are given after the species name and detailed information is given in Additional file 2: Table S1. Areas used in the biogeographic analysis: A, Africa; B, Tropical Asia; C, Japan; D, Taiwan; E, Southwest China; F, Hainan; G, most northern part of China; H, most southern part of China. The color gradients shown in the top left corner correspond to the regions. The red arrow represents the “maximum shift point”. The black shaded boxes under the time scale correspond to the flux phases of East Asian monsoon.

In total, nine clades (I-IX) were resolved in the genus (Figure 1), and for consistency they were marked in the same order as in our previous report [39]. The initial diversification of the genus *Lepisorus* dated back to around 17 Ma followed by several main cladogenese events in the period between 8 to 3 Ma (Figure 1). *Lepisorus* clades I and II showed crown-group divergences around 4 to 3 Ma that follow periods of phylogenetic stasis. The crown node age of clade V (= the former genus *Belvisia*) dated back to 10.5 Ma (Figure 1). Four clades (I to IV) had a crown group age of around or less than 6 Ma, although the stem group age dated back to more than 12 Ma.

### Ancestral area reconstruction

Southwest (SW) China, or a combined area including SW China and Japan (both 50% probability), were recovered as the putative areas of origin of *Lepisorus* (Figure 1). Subsequent range expansions had been discovered to be coinciding with the crown group diversification, e.g. clade V colonizing tropical East Asia, clade IV and VII inhabiting Taiwan and Japan. Noticeably, the species occurring in Taiwan were probably migrated from Japan (Figure 1), while the ancestors of these island clades were likely colonizers from the China mainland. The ancestral distribution ranges of clades I, III, VIII, IX were inferred to be correlated with the SW China region. The members of clade II occurred up to ca. 5,000 m. Clade I showed evidence of a rapid radiation in the Afromadagascan region starting around 1.5 Ma and the ancestors of the radiated species were originately migrated from Southwest China (Figure 1).

### Patterns of diversification

**Figure 2:**
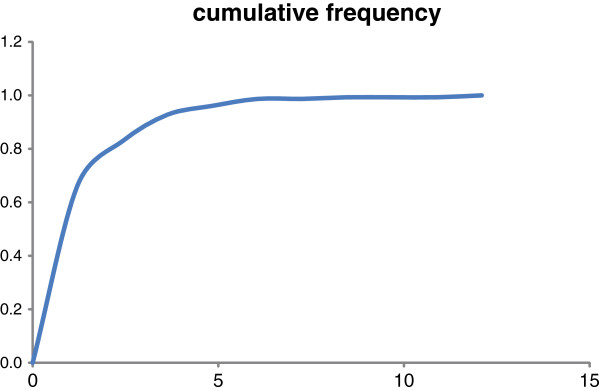
**Cumulative density of branch lengths for the phylogeny taken from a posterior sample of *Lepisorus*.** The results generated by the method of Venditti et al. (2010) correspond to variable-rates cumulative distribution function.

**Figure 3:**
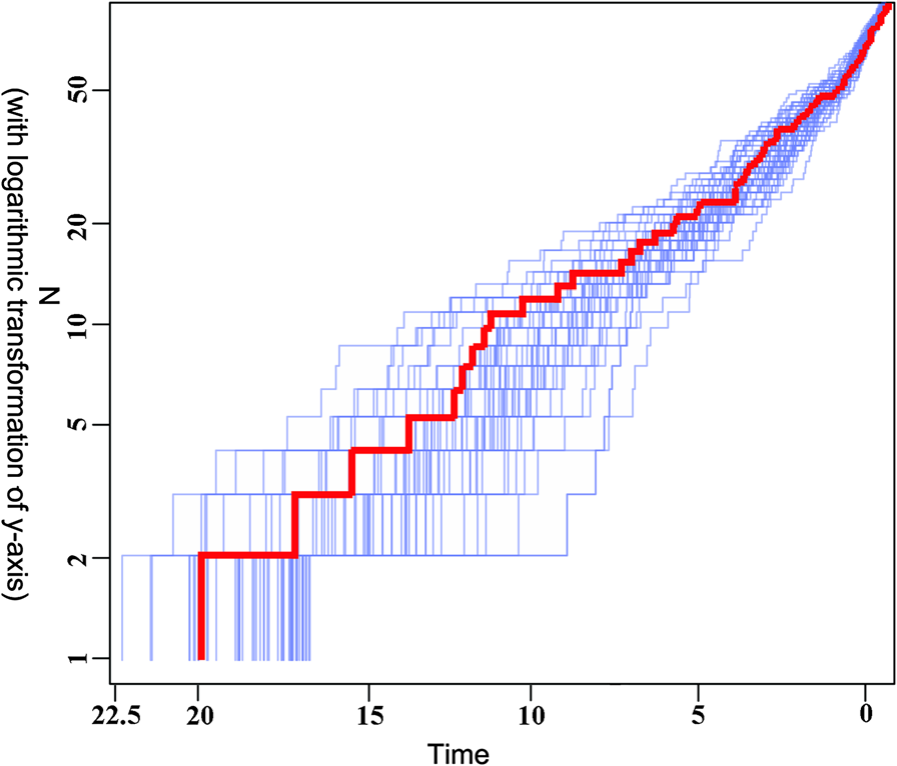
**Multiple lineage-through-time plots (MLTTP) for the genus *Lepisorus *based on 50 randomly sampled trees from the tree pool output from the Bayesian dating analysis.** The bold curve indicates the lineage-through-time plots (LTTP) for the consensus chronogram. The curve suggests two phases of shift in the diversification rate although the Constant Rate was not rejected with positive γ-value as 2.638 and p = 0.996 in LASER and APE analyses.

Exploring the divergence rates using the Constant-Rate test (CR) resulted in a positive γ-value of 2.638 with p = 0.996. This value did not reject constant rate but suggested instead an accumulation of branching events closer to the tips of the phylogeny. A Monte Carlo CR test (MCCR) indicated that the γ-value is unlikely caused by incomplete taxon sampling (Additional file 1: Figure S1). This result was inconsistent with the result recovered using the Venditti’s method [56]; the observed curve of cumulative density distribution of branch length corresponded to variable-rates cumulative distribution function (Figure 2). Lineage-through-time plots (LTTP) and multiple-lineage-through-time plots indicated an initial rapid radiation followed by a reduction of assembling diversity, which was subsequently replaced by an increase of the diversification rate towards the current time (Figure 3). The same trend was recovered in statistical analyses using Rabosky’s methods as implemented in LASER. The non-constant diversification rate was supported because the model assuming two rates fit better than the model assuming a single rate (ΔAIC_2 rates vs. 1 rate_ = 9.161). The LASER result suggested an increase of diversification rate in *Lepisorus* after its establishment as indicated by the “best shift point” with a maximum log-likelihood value of -140.4435 (Figure 1). The maximum shift-point was correlated with the first-phase increase of the diversification rate recovered in LTTP (Figure 3), while the increase of the diversification rate in the last 4 million years was not correlated to a single shift point, but it instead may be created by the accumulation of lineages in clades I, II, and IX.

**Figure 4:**
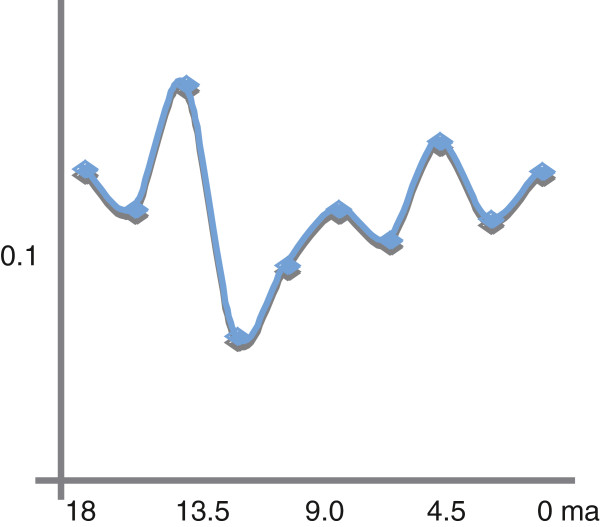
**Estimates fluctuation of diversification rates through time by considering the newly appearing species (Δn) against equal time intervals.** The calculation considers the number of newly appearing species versus the number of existing lineages at the beginning of the interval.

The hypothesis of two phases of increased diversification rate was also recovered by plotting the difference of species-birth (appearance of new species) per time intervals of 0.9 Ma. The origin of *Lepisorus* was followed by a period of increased diversification rate at about 13.5 Ma. The second phase of species-birth events was determined to date back to about 4 Ma (Figure 4).

## Discussion

### Dealing with uncertainty

Considering the results of this study, we need to take into account different kinds of uncertainty that may prevent to recover the diversification pattern of *Lepisorus*. Firstly, our dating of the lepisoroid ferns was calibrated with estimates obtained in previous analyses and thus the error of these studies holds the potential to create misleading results [33-35]. However, we lack any alternative as a result of the absence of lepisoroids in the fossil record. This is consistent with the generally poor fossil record of Polypodiaceae [34,59]. To alleviate the limitations of this approach we implemented the lognormal calibration model [60].

A further uncertainty is created by the fact that the presented estimates of divergence rates are based exclusively on extant taxa. Several authors have pointed out the limitations of divergence rate estimates applying extant taxa alone, especially in the context of estimating the extinction rate (e.g., [41-43]). Therefore, we considered our results with caution and employed several methods to explore the evidence, although Rabosky’s method provides likely the most robust test. In general, the recovered pattern points toward two periods of increased diversification rate which appears to coincide with time periods in which the climate in SW China may have considerably changed (Figures 1, 4). The rather long branches of clades (e.g. clades I to IV) showing an increased rate around 6-3 Ma may be interpreted as an indicator for replacement of older species by newly established species. Studies incorporating exclusively extant taxa are ill equipped to differentiate between the lack of speciation events along long branches or the replacement as the result of species turn-over. Our argument is based on the assumption of a relative constant speciation rate along all branches and thus long branches were not expected. Interestingly, only clade V (= the former genus *Belvisia*) shows an unambiguous signal for constant accumulation of species through time. At the same time, this clade is the only one with a mainly tropical distribution range.

Finally, the uncertainty of the taxonomy of *Lepisorus* is a major challenge. Different opinions exist about the number of species. We follow here the most recent revisions, which tend to reduce the number of recognized species [46,47]. The reduced number of accepted species is caused by the careful re-evaluation of the information provided by morphological characters and considering exhaustive number of specimens [40,46,47]. Some of the ignored species may be the result of secondary speciation via polyploidization. Allopolyploid speciation has been documented for *Lepisorus thunbergianus*[52] and our recent results suggest speciation via polyploidy as a mode of evolution in the *Lepisorus clathratus* complex [61]. Current methods of diversification rate estimates can deal exclusively with primary speciation events (cladogeneses) and the contribution of speciation events via reticulation cannot be assessed. Thus, we need to keep in mind that our study considers only speciation events that are based on cladogenese events despite the fact that many lineages of plants assemble their species diversity partly via polyploidy [53].

We also need to consider the sensitivity of the different methods employed to explore the diversification pattern. The constant-rate test of Pybus & Harvey [54] did not provide significant evidence to reject a constant diversification rate, although the positive γ-value may indicate a clustering of the branching events towards the recent. In contrast, the method of Venditti et al. [56] suggests a slow-down of the diversification rate. Both methods are rather simplistic and limit undoubtedly their explanatory power [62]. Parameter-rich approaches [55] are likely more powerful to reconstruct complex patterns of diversification rate changes such as multiple deviations from a constant rate. As a payback, these methods may be especially problematic in the context of changes affected by the extinction rate [45]. Remarkably, LASER analysis detected the early diversification of *Lepisorus* (Figure 1, red arrow) as the main shift point of the diversification rate, but it did not detect nodes corresponding to the crown groups of clades I II, IV and IX as possible shift-points. In our case, the hypothesis of multiple-changes of the diversification rates appears to be most consistent with the results recovered by different methods to infer divergence rates.

### Evidence for the impact of changing monsoon climates on fern diversity

Our results suggest two periods of increased diversification rate exist in the genus *Lepisorus*. They likely coincide with time periods suggested to be crucial phases of changes in the evolution of the East Asian monsoon (Figure 1). The initial radiation of *Lepisorus* is accordant with the proposed phase of strengthening of the East Asian monsoon at around 15 Ma, whereas the second period of diversification rate increase dates back to around 6-5 Ma, which lags behind an intensification of the East Asian monsoons [27] (Figure 1). This change of the climate is correlated with the rise of the southern part of the QTP [17,18,20,27,29]. All the clades showing the increase of diversification rates share the SW Chinese Mountains as their likely area of origin and centre of biodiversity only with the putative exception of clade II. The *L. clathratus* clade, clade II, is of particular interest because these ferns tend to grow in very high altitudes with up to 5,000 m [40]. Furthermore, clades I and II show a unique adaptation to strong seasonal climates in form of summer-green versus the ever-green leaves [39]. In these clades, the ferns undergo a dormant phase in the winter after they have shed their leaves in the late autumn.

The assembled evidences may fit best with the hypothesis of species turn-over events in which taxa with different climatic preferences were replaced with the taxa that are more adapted to the new climatic conditions. The clades with SW China as the origin centre share long branches that may reflect the replacement of previously established taxa with taxa adapted to the new climatic regime. The most outstanding exception is clade V, which corresponds to the former genus *Belvisia*. These taxa occur mainly in tropical SE Asia and thus the impact of the monsoon regimes is expected to be limited on their biodiversity. Thus, the more constant accumulation of species diversity shown by ‘*Belvisia*’ may reflect the more conservative nature of the climates in tropical SE Asia.

## Conclusions

The presented results outline the direction of the research aiming to explore the impact of the uplift of the Qinghai-Tibetan Plateau on the diversification of plant lineages occurring in SE Asia. Our results are consistent with reports on other lineages of plants such as the eudicot genera *Caragana*[16] and *Rheum*[15], the conifer genus *Juniperus*[63] or animals such as spiny frogs [10]. Most importantly, our results show evidence that the impact is not limited to plants occurring on the plateau and Asian interior areas affected by aridification. Instead, the uplift may have had a strong impact on the plant life in whole SE Asia with the exception of areas strongly influenced by maritime climates such as Japan and Taiwan. These areas may be the refuge of taxa adapted to less seasonal monsoon climates. These new findings suggest the need to study more extensively the impact of the rise of the Himalaya on the assembly and maintenance of biodiversity in SE Asia [64]. Previous studies were often focused only on genera that colonized the newly founded mountain ranges (e.g., [15,63,65]), whereas the impact on the plant diversity in the lower altitudes of SE Asia were widely ignored.

## Methods

### Datasets, taxon sampling, phylogenetic analyses

We assembled sequences of four chloroplast genome regions, *rbcL*, *rbcL-atpB* intergenic spacer sequences (IGS), *rps4* plus *rps4-trnS* IGS, and *trnL-F* region including the *trnL* intron and *trnL-trnF* IGS using sequences generated in previous studies [39,40,51]. A few additional sequences of expanded sampled species (*L. nudus* Ching, *L. nylamensis* Ching et S. K. Wu, *L. perrierianus* Ching, *L. schraderi* (Mett.) Faden, *L. rotundus* Ching and *L. vesiculari-peleaceus* (Hieron.) Pic. Serm.) were obtained using the protocols published in these studies. In total, the dataset included 65 out of the ca. 70 currently known species of *Lepisorus* plus 11 species of other lepisoroid fern genera (See Additional file 2). The taxonomy follows the most recent treatments [39,40,46-51,66]. The sampling was designed to cover more than 90% of the species diversity of *Lepisorus* and represent its geographic range without any obvious bias. All alignment were generated manually in Maclade 4.08 [67] and ambiguous regions excluded from further analyses.

Initially, the dataset was explored by carefully designed phylogenetic analyses using PAUP 4.0 [68] for maximum parsimony, PHYML 3.0 [69] for maximum likelihood and MrBayes 3.1.2 [70] for Bayesian Inference of phylogeny as described in Wang et al. [39]. The model of sequence evolution was determined using jModelTest [71] and a likelihood ratio test (LHR) was carried out to test for the presence of a molecular clock. No evidence for topological heterogeneity was discovered and all subsequent analyses were carried out with a combined dataset.

### Divergence time estimates

Bayesian approaches with uncorrelated relaxed clock model were employed to estimate the divergence times of the lepisoroid ferns with focus on the genus *Lepisorus*. The LHR test results supported the use of the relaxed clock model. All divergence time estimates were carried out using BEAST 1.6.2 ( http://beast/bioed.ac.uk) [72]. The model selected with jModelTest was implemented, but parameters were estimated simultaneously with the BEAST analyses. Several BEAST analyses were carried out and their results were summarized utilizing TRACER 1.5 ( http://beast.bio.ed.ac.uk) and TREEANNOTATOR, part of the BEAST package. Markov chains were run for 10,000,000 generations with every 10,000 generations sampled and at least a 10% burn-in phase. The final analyses were performed with a relaxed molecular clock [73], birth-death model, and calibration of the split of *Paragramma* and the remaining lepisoroid ferns [39] with a lognormal distribution model with a shift of 19.6 ma. This node age was obtained from divergence time estimate carried out with a comprehensive sampling throughout the tree of ferns [35]. Unfortunately, this study did not provide confidence intervals for nodes age estimates. The obtained age estimates were consistent with divergence time estimates of Polypodiaceae and the limited fossil record of the Polypodiaceae [33,34,59] (see also discussion). As a further confirmation, we calculated the nucleotide substitution rates for several clades and compared them with previously reported substitution rates of the chloroplast genome [74,75]. The rates were within the expected range. Shown chronograms were calculated using median clade credibility tree plus 95% confidence intervals.

### Ancestral area reconstruction

Distribution data for each species were obtained from available floristic or taxonomic treatments [39,40,46-51,66] plus information obtained from selected herbaria (BM, PE). The distribution range of *Lepisorus* was divided into eight regions of endemism (A-H) following general guidelines to define those areas. Bayesian Divergence Vicariance analyses (Bayes-DIVA) [58] were carried out using S-DIVA [76]. We used all trees in the plateau phase of the BEAST analysis (see above). The maximum area number at each node was set as two in the final analyses but alternative analyses with three or more maximal areas were carried out for comparison. We chose Bayes-DIVA considering its ability to infer ancestral biogeopraphic ranges under consideration of phylogenetic uncertainty [57,58]. The analyses were carried out with and without incorporation of the outgroup taxa. The waste majority of the outgroup taxa occurs exclusively in southwest China. Thus, the results of the analyses with and without outgroup taxa were consistent.

### Diversification rates

To investigate patterns of diversification through time, we used five approaches. First, we explored evidence for non-constant diversification rate in *Lepisorus* using the Constant Rate (CR) birth death model test [54] as implemented in several programs including Gammastatistics V.10 ( http://www.oekologie.biologie.uni-mainz.de/people/evi/home/html) and in R applications ( http://www.R-project.org): APE [77], GEIGER [78] and LASER [55]. A MCCR test using simulations of phylogenies was employed to account for the impact of missing species [54,79]. Second, we estimated the cumulative density distribution of branch length [56]. Length of the branches were detached from the calibrated consensus tree and divided into ten length classes. Frequency number of each class was calculated and plotted as cumulative frequency against branch length classes. Third, the diversification rates were visualized by generating multiple lineage-through-time plots (MLTTP) for 50 randomly selected trees from the tree pool output from BEAST using the R packages APE and GEIGER, plus lineage-through-time plots (LTTP) for the consensus chronogram with a mean node age. Fourth, we used Rabosky’s LASER software [55] in R to determine whether the observed pattern of diversification through time fits best to a simple model of a constant diversification rate or to complex models with variable diversification rates. The best fitting rate model, maximum shift points and the threshold of shift points were estimated according to the maximum log-likelihood values using Akaike Information Criterion (AIC) weights and Delta-AIC scores [55]. Fifth, we visualized changes of the diversification rate by plotting the difference of number of newly appearing species (Δn) against the setting time intervals (Δt) as 0.9 Ma. The value of Δn was then transferred into changes of diversification rate per time interval by considering the number of species at the beginning of the interval. The limitations of the outgroup sampling were considered by exploring the impact of different sampling densities. However, the sister lineage of *Lepisorus* is considerable less species-rich [51]. All the results were compared to the suggested periods of changes in the monsoon regimes and major geological events [17-20,27].

## Competing interests

The authors declare that they have no competing interests.

## Authors’ contributions

ZXC, HS and XQP designed the study. WL and ZXC collected materials. WL finished molecular experiments. WL and HS analyzed data. WL, XQP, ZXC, and HS wrote the manuscript. All authors read and approved the final manuscript.

## Supplementary Material

Additional file 1 **Figure S1.** Histogram of a null distribution of gamma statistic on incompletely sampled trees using simulation. The red arrow demonstrates the retrieved gamma value, which is outside of the 95% confidence interval, and thus indicates that the incomplete sampling does not bias the gamma statistic. Click here for file

Additional file 2 **Table S1.** Information regarding taxon names, collecting localities, voucher numbers, Genbank accession numbers and distributional ranges for taxa included in the analyses. Distributional ranges are coded as follows: A, Africa; B, Tropical Asia; C, Japan; D, Taiwan; E, Southwest China; F, Hainan; G, most northern part of China; H, most southern part of China. Click here for file
