# Supplementary figures and images for: The rise of the Himalaya enforced the diversification of SE Asian ferns by altering the monsoon regimes
Source: BMC Plant Biol. 2012 Nov 9;12:210. doi: 10.1186/1471-2229-12-210 (PMC3508991; doi:10.1186/1471-2229-12-210)

**Histogram of a null distribution of gamma statistic**

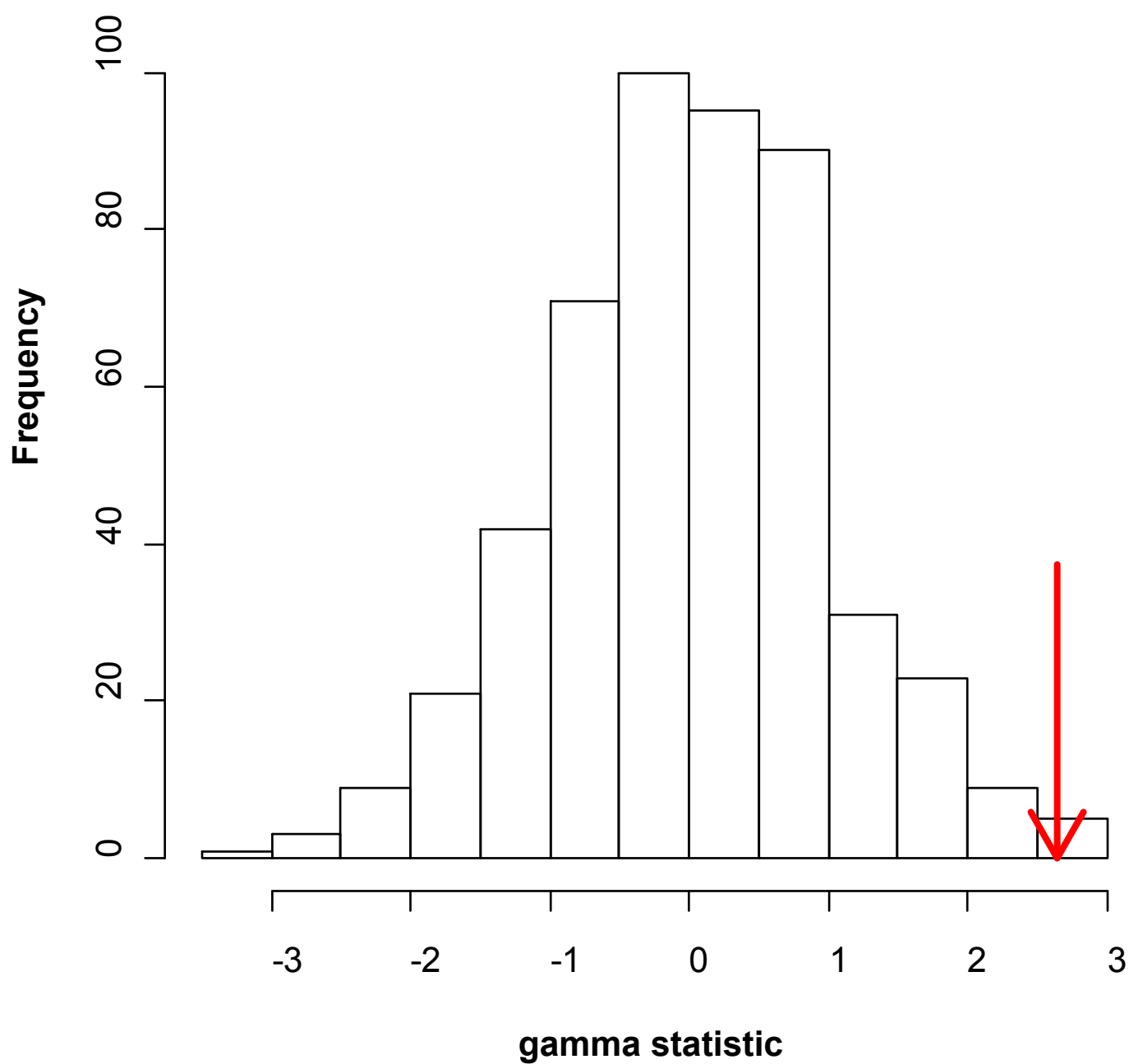

Supplement: Additional file 1 — Figure S1. Histogram of a null distribution of gamma statistic on incompletely sampled trees using simulation. The red arrow demonstrates the retrieved gamma value, which is outside of the 95% confidence interval, and thus indicates that the incomplete sampling does not bias the gamma statistic. [file 1471-2229-12-210-S1.pdf]
